# Compressive Properties of Open-Cell Al Hybrid Foams at Different Temperatures

**DOI:** 10.3390/ma10020098

**Published:** 2017-01-24

**Authors:** Jiaan Liu, Fujian Si, Xianyong Zhu, Yaohui Liu, Jiawei Zhang, Yan Liu, Chengchun Zhang

**Affiliations:** 1 Key Laboratory of Automobile Materials (Ministry of Education), College of Materials Science and Engineering, Jilin University, Changchun 130022, China; liuja@jlu.edu.cn (J.L.); 18943659380@163.com (F.S.); lyh@jlu.edu.cn (Y.L.); Zhangjw@mail.jlu.edu.cn (J.Z.); 2 Key Laboratory of Bionic Engineering (Ministry of Education), Jilin University, Changchun 130022, China; Liuyan2000@jlu.edu.cn (Y.L.); jluzcc@jlu.edu.cn (C.Z.)

**Keywords:** hybrid foams, electroless plating, compressive properties, elevated temperature

## Abstract

Hybrid Ni/Al foams were fabricated by depositing electroless Ni–P (EN) coatings on open-cell Al foam substrate to obtain enhanced mechanical properties. The microstructure, chemical components and phases of the hybrid foams were observed and analyzed by scanning electron microscopy (SEM), energy-dispersive X-ray spectroscopy (EDS) and X-ray diffraction (XRD), respectively. The mechanical properties of the foams were studied by compressive tests at different temperatures. The experiment results show that the coating is mainly composed of Ni and P elements. There was neither defect at the interface nor crack in the coatings, indicating that the EN coatings had fine adhesion to the Al substrate. The compressive strengths and energy absorption capacities of the as-received foam and hybrid foams decrease with the increasing testing temperatures, but the hybrid foams exhibit a lower decrement rate than the as-received foam. This might be attributed to the different failure mechanisms at different testing temperatures, which is conformed by fractography observation.

## 1. Introduction

Aluminum foam is a kind of material with a combination of some attractive physical and functional properties, such as low density, high energy absorption capacity, and excellent damping property [1,2].

The compressive property is one of the important properties of metal foam [2]. It is generally accepted that the compressive property relies on many factors, such as the relative density and porosity [3,4], the property of cell wall material [5,6], cell wall microstructure [7,8,9], the testing temperature [10,11,12] and surface coating on the foam [13,14,15,16,17,18,19].

Recently, some experiments showed that the coating deposited on Al foam can significantly improve its strength, stiffness energy absorption capacity and corrosion resistance [13,14,15,16,17,18,19,20]. The plasma electrolytic oxidation (PEO) treatment provides the foam with a higher compressive strength and corrosion resistance in comparison with the as-received foam because it gives a layer of protective ceramic coating on the Al struts of the foam [13,14,15]. The scanning electron microscopy (SEM) results indicated a PEO ceramic coating that mainly consists of oxides exhibiting a two-layer structure: the inner layer is characterized by fine-scale pores, while the outer layer contains much coarser pores [14]. In addition, hybrid metal foams can be created by reinforcing open-cell Al foam with metal base coatings (Cu and Ni) using electrodeposition. These coatings improve some mechanical properties of open-cell Al foam by forming Ni/Al or Cu/Al hybrid foams, consisting of Al struts coated with nanocrystalline Ni or Cu. It was illustrated that the hard coating on the Al struts enhanced the strength and energy absorption capacity of the hybrid foams by strengthening the Al struts against bending and buckling [16,17,18,19]. The analysis results from SEM and X-ray diffraction (XRD) showed that a proper annealing can significantly increase the energy absorption capacity of Al/Cu hybrid foams by changing the microstructure and average crystallite size. However, excessive annealing results in a number of intermetallic compounds at the Al/Cu interface, which reduces the performance of the hybrid foams [19].

However, these experiments mentioned above mainly described the mechanical properties of the hybrid foams at room temperature, but limited experimental data at elevated temperature were available. It is generally accepted that the mechanical properties of metal foam are strongly dependent on the testing temperature [10,11,12].

Therefore, in the present work, the compressive property and energy absorption capacity of as-received Al foam and Ni/Al hybrid foams were studied at different testing temperatures. The failure mechanisms of the Ni/Al hybrid foams at different testing temperatures were also investigated by means of SEM observation and energy-dispersive X-ray spectroscopy (EDS) analysis.

## 2. Experimental Section

### 2.1. Preparation

The pure aluminum foams were fabricated by a pressure-infiltrated process. The details of preparation process are given by our previous experiment [21].

The porosity of the Al foam were calculated using the following equation:

*P* = (1 − ρ^*^/ρ_s_) × 100% (1)

where *P* is the porosity of the foam, ρ^*^ and ρ_s_ the density of the foam and the metal matrix, respectively, and ρ^*^/ρ_s_, which is called the relative density of the foam, indicates the ratio of the density of the foam to the density of the matrix material.

The density of foam was calculated by weighing and measuring each individually. The density of matrix material was evaluated by density of pure aluminum.

The foams were cut into several foam specimens. Before electroless plating, the foam specimen needs to be pretreated because there are plenty of oxides on the surface of the aluminum foam. The foam specimens were ultrasonically cleaned in acetone at first, followed by alkaline cleaning and the acid pickling process. The pretreatment process gives the foam specimen a relatively clean surface. After pretreatment, the foam specimens were electroless deposited. The main chemicals of the bath were listed in Table 1. The details for the electroless plating process were described in reference [22].

### 2.2. Compression Test

Specimens were prepared for compressive test at a nominal strain rate of ~10^−3^/s. For the high temperature test, a servo-hydraulic machine (Instron series, Instron Corporation, Shanghai, China) was used at a nominal strain rate of ~10^−3^/s. The machine was instrumented with an ambient chamber to maintain the testing temperatures within ±5 °C. The temperatures of ambient chamber were set up at 100 and 200 °C. Prior to a compressive test, each foam specimen was heated to the desired temperature and left in the ambient chamber for more than 20 min to achieve temperature stability. Afterwards, the compressive test was employed. In the present study, the crosshead displacements were recorded to calculate the compressive strain, and this measured method was applied on Ni/Al hybrid foams in a recent study reported by Bouwhuis [17]. The obtained data were used for drawing the compressive engineering stress–strain curves.

The energy absorption capacity of metallic foams, *W*, can be obtained from the area under the stress–strain curve up to a certain strain [1,2], namely:

(2) $$W=\int_{0}^{\varepsilon }\sigma d\varepsilon$$

where ε is the compressive strain; and σ is the compressive stress.

The energy absorption efficiency, *I*, can be calculated using the following equation [1,2]:

(3) $$I=\frac{1}{\sigma \max\varepsilon }\int_{0}^{\varepsilon }\sigma d\varepsilon$$

where σ_max_ is the maximal stress in the stress–strain curve.

### 2.3. Material Characterization

The microstructures of the foams were analyzed by means of scanning electron microscopy (SEM, Model JSM-5310, JEOL, Mitaka, Japan and Model EVO18 ZEISS, Zeiss GmbH, Jena, Germany) with an energy-dispersive spectrum analyzer (EDS, Model Link-Isis, Oxford Comp., Abingdon, UK).

Polished cross sections of foam specimens were examined with SEM and the measured coating thicknesses were averaged based on more than 15 measurements at different locations throughout foam specimens to achieve result repeatability.

The phases of the substrate and coating were analyzed by X-ray diffractometer (XRD, D/Max2500PC, Rigaku Corporation, Osaka, Japan). The radiation source is Cu Kα. The scanning angular scope is from 20° to 80°, and the scanning speed is 4°/s.

In order to clarify the failure mechanism of the Ni/Al hybrid foams at different testing temperatures, the fractography observation was made. The foam specimens were observed using SEM after a fixed compressive strain of ~0.3.

## 3. Results and Discussion

### 3.1. Surface Morphology and Thickness of the Coatings

**Figure 1:**
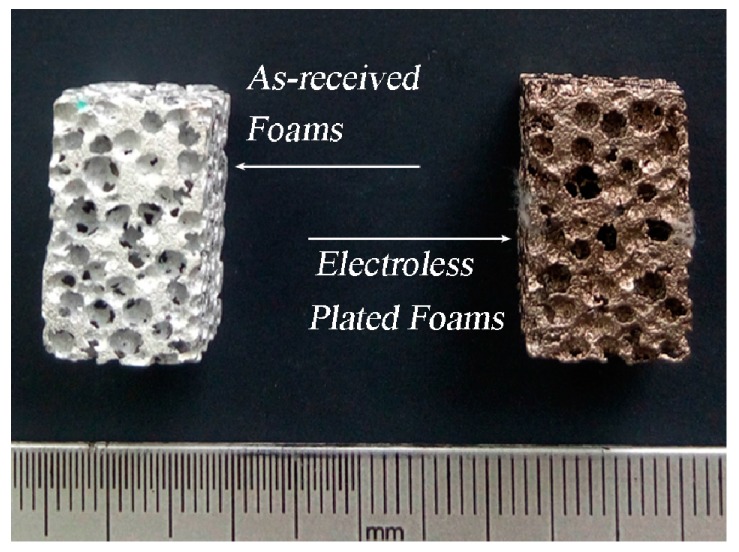
The macroscopic morphology of: (**left**) as-received foam; and (**right**) electroless plated foam (Ni/Al hybrid foams).

**Figure 2:**
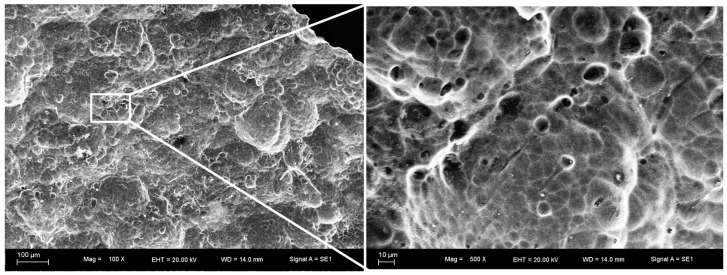
The surface topographies of as-received foam.

**Figure 3:**
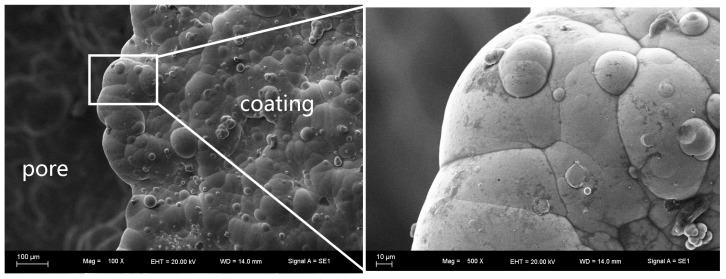
The surface topographies of electroless Ni–P (EN) coating.

The as-received foam and electroless plated foam with porosities of ~65% are shown in Figure 1. The surface topographies of the as-received foam and electroless Ni–P (EN) coatings are shown in Figure 2 and Figure 3, respectively. It can be observed that the surface of as-received foam contained plenty of irregular scalloped pits after the pretreatment. The electroless plating produces a rough coating surface. This coating consists of nodule morphology. The size of the nodules is not uniform and it varies from 10 to 30 μm. Some of the nodules overlap on each other. However, the coating is dense and microcrack was not observed on the coating surface. The lack of microcrack is favored for the mechanical property of the coating.

The Ni–P coating makes a large mass change because the density of the Ni–P alloys is ~8.9 g/cm^3^, as compared to 2.7 g/cm^3^ for pure Al. The mass varies from 2.97 g for the as-received foam to 4.23 g for the Ni/Al hybrid foams. It indicated that the density of the Ni/Al hybrid foams is much higher than that of as-received foam.

**Figure 4:**
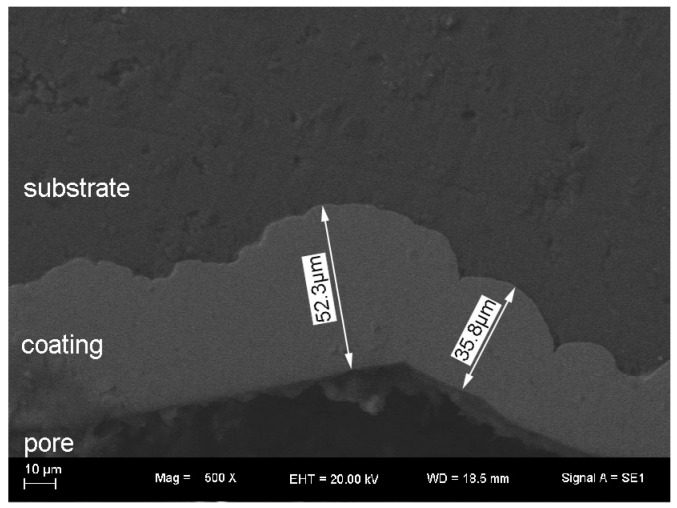
The cross section of electroless plating coating.

The cross section of the coating is shown in Figure 4. There is a distinct interface between the substrate and coating. The bonding is fine on the substrate/coating interface and SEM examination did not show the microcrack in the inner of the coating as well.

The average thickness of the EN coating is ~36.5 μm from statistical results. It is generally accepted that the EN coatings deposited by electroless plating technology have a relative uniform thickness. However, it is noted that varieties in thickness were found in the present study. The different thicknesses of EN coating on different locations were typically shown in Figure 4, which exhibits that the thicknesses are roughly ~52.3 μm and ~35.8 μm on neighbor locations. A potential reason is that there are a number of oxides on the surface of the foam, and, therefore, the surface of the foam is rather rough as compared to that of bulk metal. After pretreatment and electroplating, the nodule size of EN coating is not uniform in size, and therefore it increases the diversity of thicknesses. In addition, owing to the complex structure of Al foams, it causes a limited mass transportation of the solution from the outer to the inner part of the foam. This is not confirmed and it remains to be proven. The related factor and reason for that will be investigated in further study.

### 3.2. Chemical Composition and Phase Composition of Coatings

**Figure 5:**
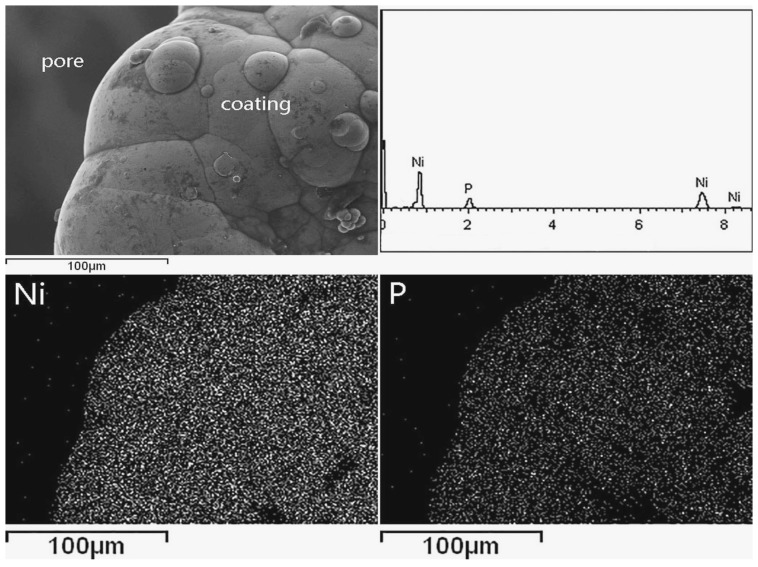
The element distribution of EN coating surface.

**Figure 6:**
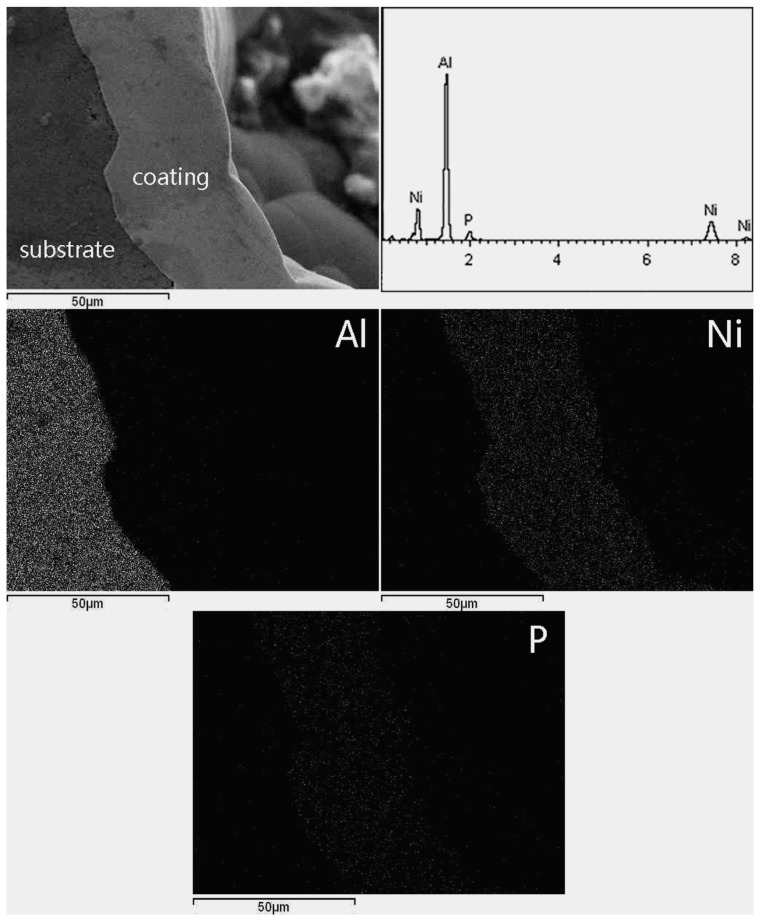
The element distribution of EN coating.

The EDS analysis results on surface and cross section of EN coating are shown in Figure 5 and Figure 6, respectively. The horizontal and vertical axes were two-dimensional locations. They illustrated that the coating is mainly composed of the two compositions, i.e., Ni and P, and the content of the P element is about 10.12 wt %.

**Figure 7:**
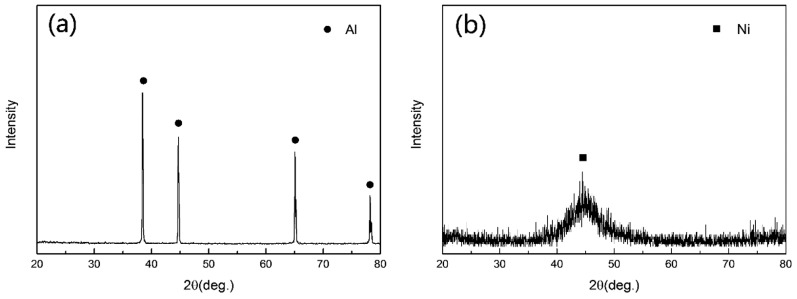
The X-ray diffraction (XRD) patterns of: (**a**) as-received foam and (**b**) hybrid foams.

The XRD patterns of as-received foam and hybrid foams are shown in Figure 7a,b, respectively. It can be seen that the XRD pattern exhibits only a single broad peak at about 45°, which corresponds to the (111) plane of the face-centered-cubic (FCC) phase of nickel (2θ = 44.8°). Theoretically, a disorder in the atomic arrangement manifests itself as a broad peak in XRD patterns. The content of the phosphorus element plays a key role in determining the structure of the electroless plating coatings [23,24]. Since the required phosphorus segregation is large, it prevents the nucleation of the FCC Ni phase, which leads to an amorphous structure. Therefore, when the phosphorus content is high, the EN coatings are mainly composed of amorphous structure. On the contrary, the EN coatings containing low phosphorus content are mainly composed of nanocrystalline structure [23]. The EN coatings with medium phosphorus content have semi-amorphous or amorphous structures [24]. In this study, the Ni–P coatings contain ~10.0 wt % phosphorus. Therefore, it can be inferred that the coating might be in an amorphous phase.

### 3.3. Compressive Properties of the Foam at Different Temperatures

**Figure 8:**
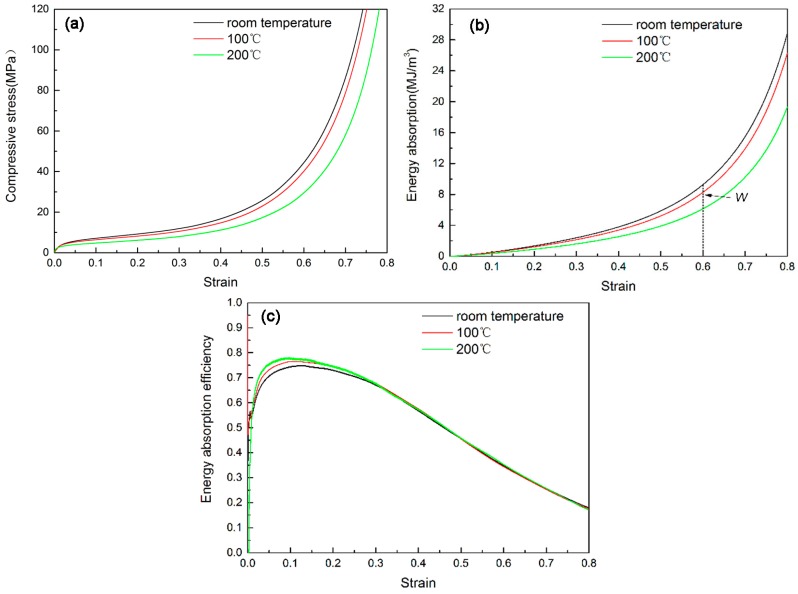
The compressive properties of the as-received foam at different temperatures: (**a**) compressive curves; (**b**) energy absorption; and (**c**) energy absorption efficiency.

The compressive responses of as-received aluminum foam at different temperatures are shown in Figure 8. It can be seen from Figure 8a that all stress–strain curves exhibit three distinct regions [1,2]: a linear-elastic region, a plateau region and a densification region. Smoothly increasing stress in the plateau region was observed in the stress–strain response of the foam, and there was no significant drop in stress after onset of the plateau region. When the testing temperature increases, the compressive stress of the foam reduces. Compared with the compression strength at room temperature, the compressive strength at 100 °C has a slight decrease, but the compressive strength at 200 °C has a dramatic reduction due to the softening effect to the foam matrix. In addition, note that the densification point extends with increasing testing temperature.

**Figure 9:**
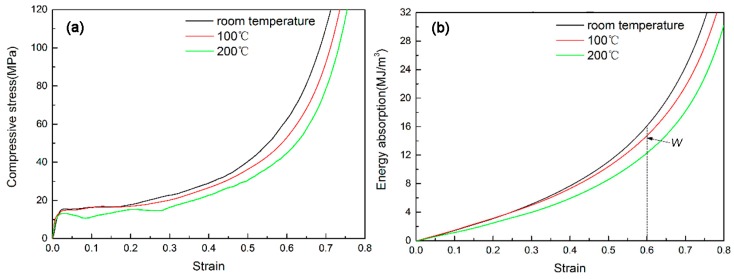

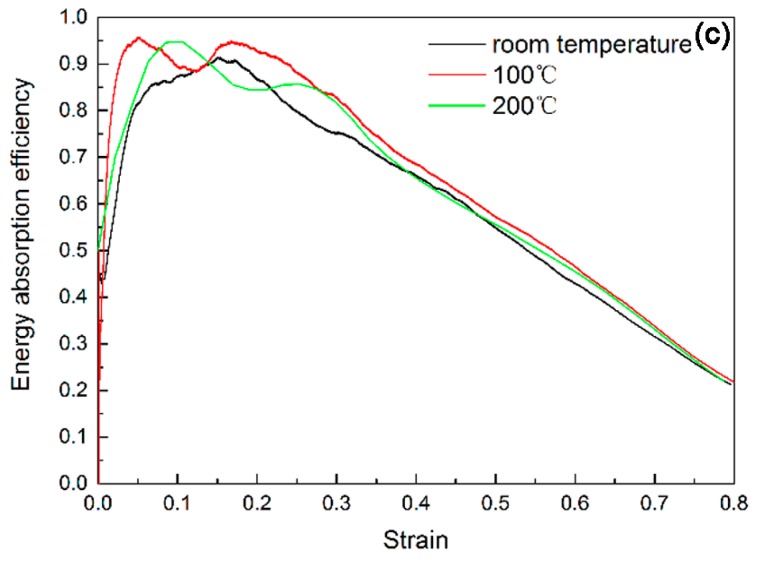
The compressive properties of the hybrid foams at different temperatures: (**a**) compressive curves; (**b**) energy absorption and; (**c**) energy absorption efficiency.

The compressive responses of hybrid foams at different temperatures are shown in Figure 9. Similar to the as-received foam, the hybrid foams also exhibit a decreasing compressive strength with rising testing temperature. The temperature makes a reducing effect on energy absorption capacity as well.

There are differences in compressive responses between two types of foams at the testing temperature of 200 °C. The as-received foam is characterized by smooth and steadily rising stress in the plateau region. In contrast, the hybrid foams exhibit stress fluctuation in the plateau region. It might be due to structure and phase transformation behavior of electroless Ni–P coatings when the coatings were heat-treated at 200 °C. The earlier research found that the respective XRD presents a sharp peak corresponding to heat-treated crystalline Ni (111) when the electroless Ni coatings were heat treated at 200 °C, which indicates the crystallization of the phase [25]. It is also thought that the short-range atomic movements resulted in the growth of larger grains at 200 °C [26].

The energy absorption capacity of the foam reduces rapidly with the increasing temperature. However, the hybrid foams show a stronger energy absorption capacity than as-received foam even at 200 °C. Therefore, EN coating provides a good strengthening effect on Al struts to resist the heat.

The decreased rates of energy absorption capacity at a strain of 0.6 for the hybrid foams are ~8.5% and ~23.7% when the temperature are 100 and 200 °C, respectively. In contrast, these values for the as-received foam are ~10.7% and ~33%, respectively. Therefore, it can be concluded that the hybrid foams exhibit a lower decrement rate compared with the as-received foam at the elevated testing temperature, especially at 200 °C. Therefore, it can be inferred that there might be different failure mechanisms for hybrid foams when the testing temperature is increasing.

### 3.4. Failure Mechanism Analysis

**Figure 10:**
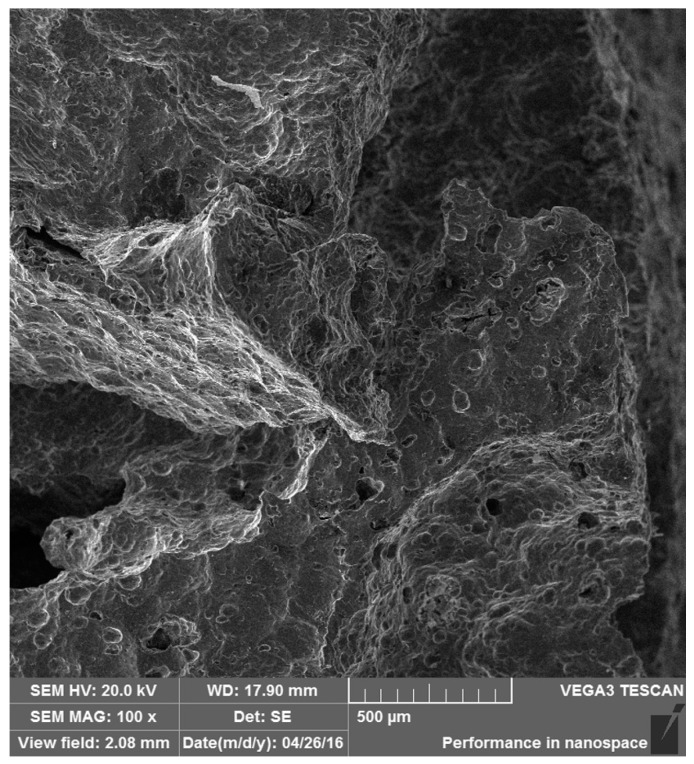
Failure of as-received foam at room temperature.

**Figure 11:**
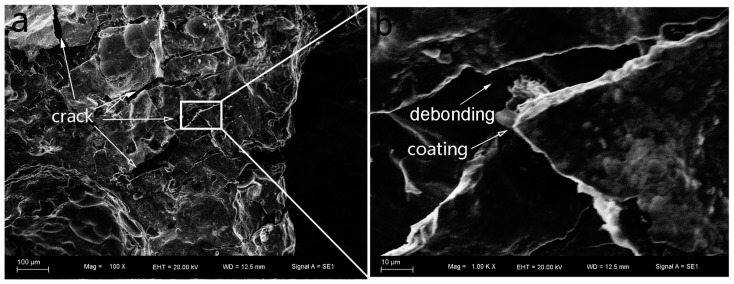
Failure of Ni/Al hybrid foams at room temperature: (**a**) 100× and (**b**) 1000×.

**Figure 12:**
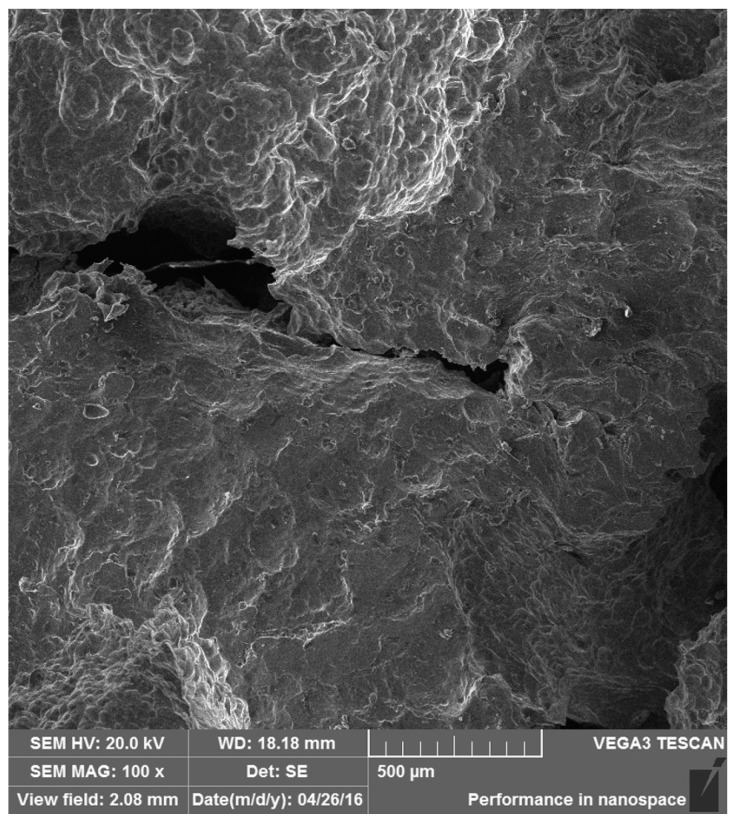
The failure of as-received foam at 200 °C.

**Figure 13:**
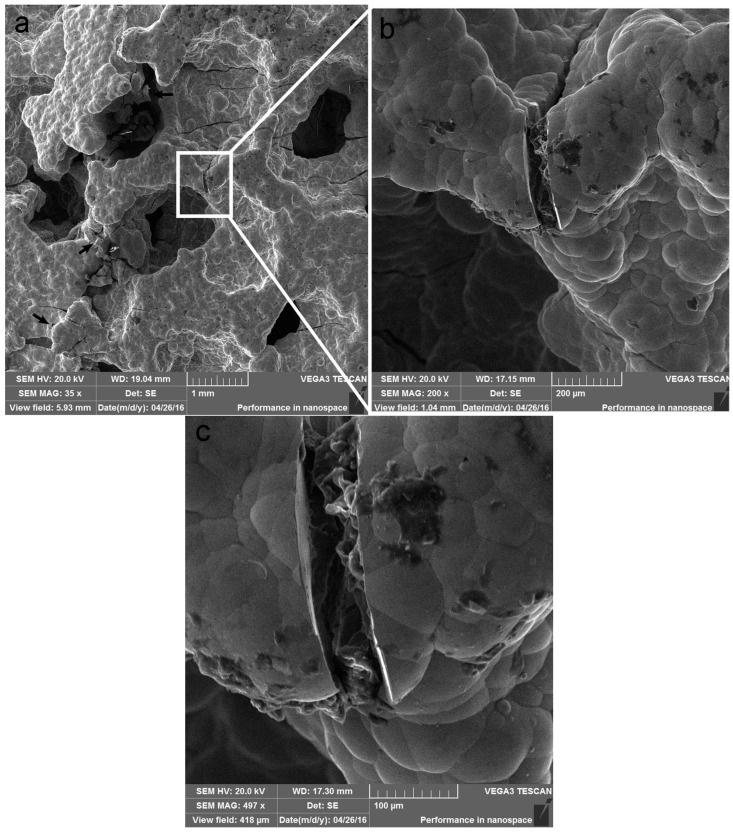
Failure of Ni/Al hybrid foams at 200 °C: (**a**) 35×; (**b**) 200× and (**c**) 497×.

To clarify the different failure mechanisms of the as-received foam and Ni/Al hybrid foams between room temperature and 200 °C, the fractography observation after a fixed compressive strain ~0.3 was made. Figure 10 and Figure 11 exhibit the fractographies for two types of foams at room temperature. And Figure 12 and Figure 13 exhibit the fractographies at 200 °C.

The failures of the as-received foam at room temperature and 200 °C are shown in Figure 10 and Figure 12, respectively. The compression direction is along vertical axes in the figures. Both the bending/buckling and fracture can be found at room temperature and 200 °C. It is indicated that the failure mechanisms of as-received foam at different temperatures are similar, i.e., bending/buckling and fracture of the foam strut.

The failures for the Ni/Al hybrid foams at room temperature and 200 °C are shown in Figure 11 and Figure 13, respectively. It is obvious that there are several broken deposit fragments and some interface debondings, as shown by the arrow in Figure 11b. Several large cracks are straight, indicating a transcrystalline rupture of the Ni deposit. The debonding of the interface between the coating and Al substrate can also be seen, which is supported by the high magnification image (Figure 11b). It is obvious from Figure 13 that some cracks can be found on the coating. Higher magnification (shown in Figure 13c) exhibits a crush of coating, but the debonding between the coating and Al substrate is not significant. More SEM observations showed that there are less debonded interfaces at 200 °C than that at room temperature. This results in increasing tolerance of Al strut to heat, and, therefore, the EN coatings still continued protecting the Al strut against the bending and buckling and reduced the softening effect on the foam matrix.

## 4. Conclusions

In this study, the electroless plating technology was applied to create the EN coatings on open-cell aluminum foam, forming Ni/Al hybrid foams. The compressive strength and energy absorption of the as-received foam and Ni/Al hybrid foams decrease with the increasing testing temperatures. However, the Ni/Al hybrid foams exhibit a lower decrement rate than the as-received foam. A main reason is that there are different failure mechanisms of Ni/Al hybrid foams at different testing temperatures.

## Figures and Tables

**Table 1:** The main composition of bath.

Electrolyte	Concentration (g/L)	Electrolyte	Concentration (g/L)
NiSO_4_·6H_2_O	25	C_3_H_6_O_3_	30
NaH_2_PO_2_·H_2_O	30	C_3_H_6_O_2_	2.2
CH_3_COONa	20	-	-
